# Development and Perfection of Marine-Based Insecticide Biofilm for Pea Seed Protection: Experimental and Computational Approaches

**DOI:** 10.3390/molecules30071621

**Published:** 2025-04-04

**Authors:** Fatouma Mohamed Abdoul-Latif, My Ismail El Mhamdi, Ayoub Ainane, Ali Merito Ali, Khadija Oumaskour, Sanaa Cherroud, Stefano Cacciatore, Tarik Ainane

**Affiliations:** 1 Medicinal Research Institute, Centre d’Etudes et de Recherche de Djibouti, IRM-CERD, Route de l’Aéroport, Haramous B.P. 486, Djibouti City 77101, Djibouti; alimerito@hotmail.fr; 2 Superior School of Technology of Khenifra (EST-Khenifra), University of Sultan Moulay Slimane, BP 170, Khenifra 54000, Morocco; moulayismail.elmhamdi@usms.ma (M.I.E.M.); a.ainane@usms.ma (A.A.); k.oumaskour@gmail.com (K.O.); s.cherroud@usms.ma (S.C.); 3 Bioinformatics Unit, International Centre for Genetic Engineering and Biotechnology (ICGEB), Cape Town 7925, South Africa; stefano.cacciatore@icgeb.org

**Keywords:** composite biofilm, insecticide, marine products, protection, pea seeds, calothrixin A, biodegradability

## Abstract

This work aims to develop an insecticidal biofilm based on Calothrixin A, collagen, and chitosan for the protection of pea seeds. The main objective is to improve the ingredient concentrations maximizing the insecticidal activity of the biofilm and to study the desorption of Calothrixin A according to the diffusion parameters. Eight biofilm formulations were prepared with different concentrations of the components and tested on *Sitona lineatus* and *Bruchus pisorum*. The results show that a high concentration of Calothrixin A tended to increase insecticidal activity, although this increase was not always significant, while a higher concentration of collagen and chitosan reduced insecticidal activity, probably by limiting the diffusion of the active ingredient. The prediction models for insecticidal activity showed that the interaction of the factors had no significant impact on the responses, but the model for *Sitona lineatus* presented better accuracy. The diffusion tests revealed that the CB3C-5 biofilm, with high diffusion parameters, correlated with insecticidal activity. The characterization of the CB3C-5 biofilm showed adequate physical, mechanical, thermal, and structural properties for agricultural seed storage application. Moreover, the computational approach showed that Calothrixin A interacts more efficiently with the OR5-Orco complex than with the small OBP, disrupting the olfactory detection of insects. This mechanism highlights the targeting of the olfactory complex as a potential strategy to control insect pests. This research contributes to the understanding of the role of marine-based biofilms for seed protection and opens perspectives for the development of ecological solutions against insect pests, particularly in the field of sustainable agriculture.

## 1. Introduction

**Figure 1:**
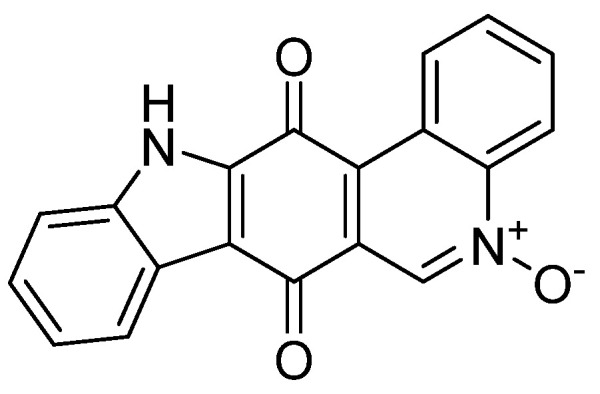
Calothrixin A.

The field of food packaging has undergone a revolution in recent decades, aimed at extending the shelf life of products while preserving their organoleptic qualities [1]. However, the rise in plastic packaging has led to serious environmental problems, including the pollution of oceans and soils, which have been exacerbated by the proliferation of single-use plastics [2]. This situation has raised growing concerns among consumers about food safety and potential risks to human health, highlighting the urgent need to find sustainable and environmentally friendly alternatives [3]. It is in this context that research has focused on biodegradable and edible packaging films based on biopolymers, such as polysaccharides (starch, agar, cellulose, chitosan), proteins (collagen, gelatin, soy), and lipids. These materials offer a potential way to minimize the ecological footprint while maintaining food safety standards [4]. Insecticidal properties of packaging biofilms have also become a growing area of interest, particularly in agricultural applications [5]. Pests, which infest seeds during storage, represent a major problem, causing significant economic losses for agricultural producers. To address this issue, many researchers have explored the integration of insecticidal properties into biopolymer-based films [6]. These composite biofilms, which are both biodegradable and active against pests, have the potential to offer a more environmentally friendly solution, compared to conventional chemical treatments used in the agricultural industry [7]. In parallel, marine natural products, such as Calothrixin A (Figure 1), have attracted particular attention for their insecticidal properties [8]. This compound, of marine origin, is recognized for its effectiveness in repelling various types of pests while having a low ecological impact. Marine environments, rich in biodiversity, offer a wide range of biological products with unique properties, which has led to notable advances in drug development, as well as in the formulation of ecological plant protection products. Marine products have therefore been identified as potential candidates for the development of natural insecticidal solutions, particularly suitable for seed protection applications [9,10].

Recent research has identified various marine compounds with promising insecticidal activities, including terpenes, flavonoids, alkaloids, and peptides [11,12,13,14]. However, the integration of these compounds into biodegradable packaging biofilms remains a relatively unexplored avenue, particularly for specific applications such as seed protection during storage [15]. The need for innovative solutions is therefore still pressing to fill the gap in effective, biodegradable, and environmentally friendly products capable of meeting the current challenges of sustainable agriculture.

Despite promising advances in the field of biodegradable and insecticidal packaging biofilms, several limitations remain [16]. Indeed, existing composite biofilms often present defects in terms of biodegradability, stability, and efficacy. Although work has focused on the integration of natural active ingredients into biofilms, their application for seed protection, particularly small seeds such as peas, remains understudied [17,18,19]. In addition, the accurate modeling of the transfer of active compounds in the seed storage environment is still poorly developed, which limits the understanding of the release mechanisms and efficacy of composite biofilms. There is also a lack of in-depth research on the interaction of marine biopolymers with insecticidal compounds, which prevents the accurate prediction of their long-term effects [20]. The main challenge of this study lay in the need to develop an innovative solution for the protection of pea seeds, a key crop in many agricultural regions. During the storage phase, seeds can be subject to insect infestations, which leads not only to economic losses but also to a degradation of seed quality. These losses are particularly critical for small producers, whose profitability depends on seed quality and their ability to prevent infestations. The use of chemical insecticides is often inappropriate due to their toxicity and environmental impact. Thus, a solution based on marine biopolymers, incorporating natural compounds such as Calothrixin A, collagen, and chitosan could meet this need. These materials offer interesting perspectives to the design of biodegradable biofilms with a dual function while respecting ecological imperatives: seed conservation and protection against pests.

The main objective of this study is to develop a composite biofilm based on marine products, integrating compounds such as Calothrixin A, collagen, and chitosan, in order to offer an effective solution for the protection of pea seeds against pests during storage. To do this, this study focused on optimizing the formulation of these biofilms by adjusting the preparation conditions in order to increase their insecticidal efficacy while ensuring their biodegradability. The biofilms were then characterized through various technical analyses, such as electron microscopy and spectroscopy, to assess their structure, composition, and mechanical properties. Another area of research concerned the modeling of the transfer of active compounds, by studying the release of Calothrixin A and its interaction with the storage environment. Finally, computational simulations made it possible to analyze the mechanisms of action of Calothrixin A at the molecular level, by studying its biological interactions and the targeted biological pathways, in order to better understand its effectiveness as a natural and sustainable insecticide.

## 2. Results

### 2.1. Optimization of Biofilm Preparation

The optimization of the biofilm preparation was carried out in the framework of the development of an insecticide biofilm based on marine products (Calothrixin A, collagen and chitosan) for the protection of pea seeds. An optimization of the preparation of composite biofilms (CB3C) was carried out by adjusting levels −1 and +1 for each product for a total of eight composite biofilms. After each preparation, insecticide tests were carried out on *Sitona lineatus* and *Bruchus pisorum* (pea seeds).

Table 1 presents the data from the experimental design, used to study the effect of the three factors on two distinct responses, namely the pLD50 values of the insecticidal activities. Regarding the pLD50 of *Sitona lineatus* and *Bruchus pisorum*, the highest values (4.56 and 4.59, respectively) were observed when the concentration of Calothrixin A was high, while those of collagen and chitosan were low (CB3C-5). Calothrixin A is considered a key factor increasing the insecticidal activity, while an increase in collagen and chitosan could reduce this activity [21,22,23].

Table 2 presents the two mathematical models used to predict the pLD50 of *Sitona lineatus* and *Bruchus pisorum* based on factors and their interactions. Both models for pLD50 (SL) and pLD50 (BP) showed that the studied factors, as well as their interactions, do not have a significant impact on the measured responses. In other words, the *p*-values of both models are too high to indicate a significant influence of these factors on the responses. Although the F values are relatively high for *Sitona lineatus* (85.22) and moderate for *Bruchus pisorum* (4.68), the *p*-values indicate that these values are not statistically significant. This means that the observed differences are likely due to random variation in the data, rather than real effects of factors on the responses [24].

The statistical parameters of the pLD50 for *Sitona lineatus* (SL) and *Bruchus pisorum* (BP) are detailed in Table 3. For *Sitona lineatus* (SL), the statistical parameters show that the model is of high quality, with excellent accuracy, good predictive power, and low data dispersion around the mean. *Sitona lineatus* (SL) appears to be well explained by the model, with reliable fit and prediction. For *Bruchus pisorum* (BP), although the model explains a large proportion of the variance (with a good R^2^), the adequate precision and predicted R^2^ are lower, suggesting that the model for BP has less reliable predictive power and may not fit the data well. The greater dispersion in BP values (indicated by the standard deviation and coefficient of variation) also suggests greater variability in the response for BP compared to SL.

**Figure 2:**
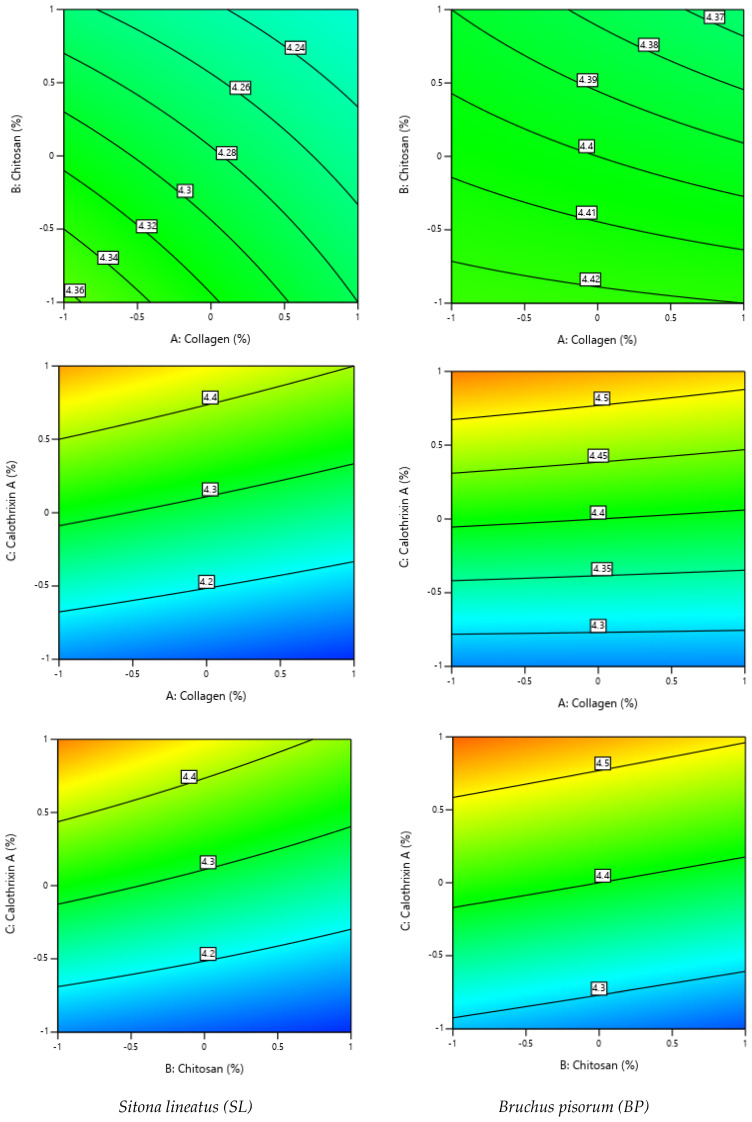
Two-dimensional graphical representations of contour plots for two factors to predict pLD50.

Figure 2 presents the contour plots, which are 2D graphical representations used to visualize the effect of each pair of factors among the three studied, to predict insecticidal activity against *Sitona lineatus* (SL) and *Bruchus pisorum* (BP).

### 2.2. Desorption of Calothrixin A: Diffusion and Insecticidal Activity

The study of the desorption of Calothrixin A from the biofilm was carried out using Fick’s second law [25]. This analysis included the examination of the diffusion parameters, determined by the diffusion coefficient (D), the transfer rate (K), and the desorption flux (F), as well as a correlation between these parameters and the insecticidal activity, measured by the pLD50 (SL) and pLD50 (BP).

The results of the diffusion parameters, presented in Table 4, show the values for different formulations of the biofilms (CB3C-1 to CB3C-8). The diffusion coefficient (D), representing the rate at which Calothrixin A diffuses into the biofilm, reveals that CB3C-5 has the highest value (3.82), indicating rapid diffusion. The transfer rate (K), related to the adsorption capacity of the biofilm, shows that CB3C-5, with a value of 2.62, suggests a high retention of Calothrixin A. Finally, the desorption flux (F), which indicates the release rate of Calothrixin A, shows that CB3C-5 also has the highest value (3.00), indicating a rapid release of the active substance.

The correlation matrix, presented in Table 5, reveals a strong positive correlation between the diffusion parameters (D, K, F) and the insecticidal activity measured by pLD50 (SL) and pLD50 (BP). A correlation of 0.829 between D and pLD50 (SL) shows that a rapid diffusion of Calothrixin A in the biofilm is associated with higher insecticidal activity. Similarly, the correlation between K and pLD50 (SL) (0.828) suggests that biofilms with high adsorption capacity enhance insecticidal efficacy. The desorption flux (F) is also highly correlated with insecticidal activity, with correlations of 0.755 for pLD50 (SL) and 0.867 for pLD50 (BP), indicating that the rate of release of the substance is a determining factor for the efficacy of the biofilm.

**Figure 3:**
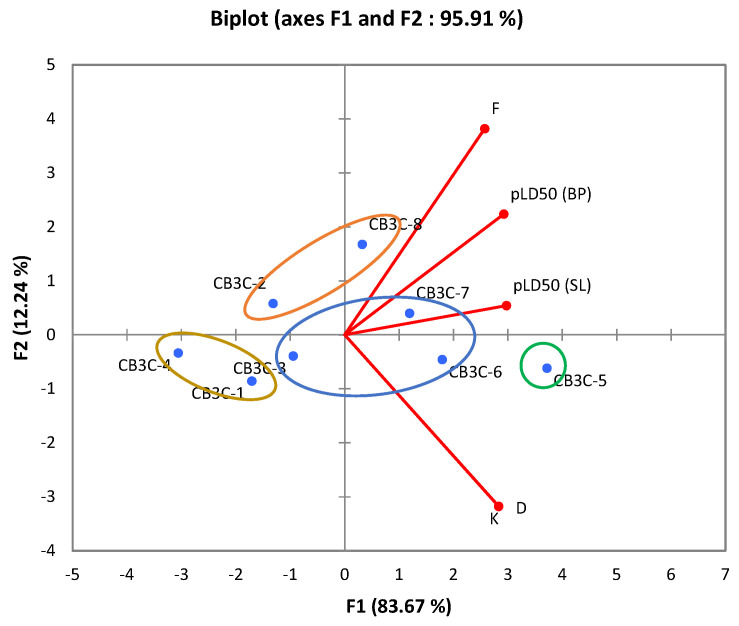
Principal component analysis between diffusion parameters and insecticidal activity of Calothrixin A.

These results show that the diffusion parameters (D, K, F) strongly influence insecticidal activity. A biofilm with high values for these three parameters allows rapid diffusion, optimal retention, and rapid release of Calothrixin A, which optimizes insecticidal efficacy. The CB3C-5 biofilm, with its high values for D, K and F, was found to be the most effective, which was confirmed by the pLD50 results. Principal component analysis (Figure 3) also confirmed this relationship between diffusion parameters and insecticidal activity for the CB3C-5 biofilm, showing a strong direct correlation with both strains tested.

### 2.3. Characterization of the CB3C-5 Biofilm

The development of the CB3C-5 biofilm was subjected to a series of analyses to evaluate its physical, mechanical, thermal, and structural properties, in the context of its potential use for the protection of pea seeds against *Sitona lineatus* and *Bruchus pisorum*. These evaluations allowed us to determine the performance of the biofilm under different environmental conditions, which is essential for its application in agriculture.

The physical and mechanical properties of the CB3C-5 biofilm were measured using standardized tests (Table 6). The thickness of the biofilm was determined to be 0.12 ± 0.02 mm, indicating a relatively thin and homogeneous structure [26]. Regarding moisture absorption, it was measured at 3.9 ± 0.2%, revealing a moderate capacity to absorb moisture. This capacity is basic to maintain the flexibility of the biofilm without it drying out or becoming brittle. Moderate moisture absorption helps prevent desiccation problems while maintaining flexibility, which is particularly important in environments with fluctuating climates and varying humidity [27]. The water solubility of the biofilm was measured to be 26.5 ± 2.4%, indicating that the biofilm is partially soluble in water. This moderate solubility may influence the stability of the biofilm in aqueous environments (contact with water) [28]. Opacity was measured to be 1.1 ± 0.1 A mm^−1^, indicating that the biofilm allows some light transmission. This characteristic is particularly important in seed storage, where protection from excessive light exposure is required while allowing humidity and temperature control [29]. The water vapor permeability (WVP) of the biofilm was measured at 1.8 ± 0.3 g m^−1^ Pa^−1^ s^−1^, allowing for effective moisture management while maintaining the necessary balance for seed protection [30]. Finally, the tensile strength of the biofilm was measured at 17.4 ± 1.5 MPa, indicating that the biofilm is relatively robust and able to withstand mechanical stress without tearing, which is essential for use in applications requiring durability, such as seed protection [31].

**Figure 4:**
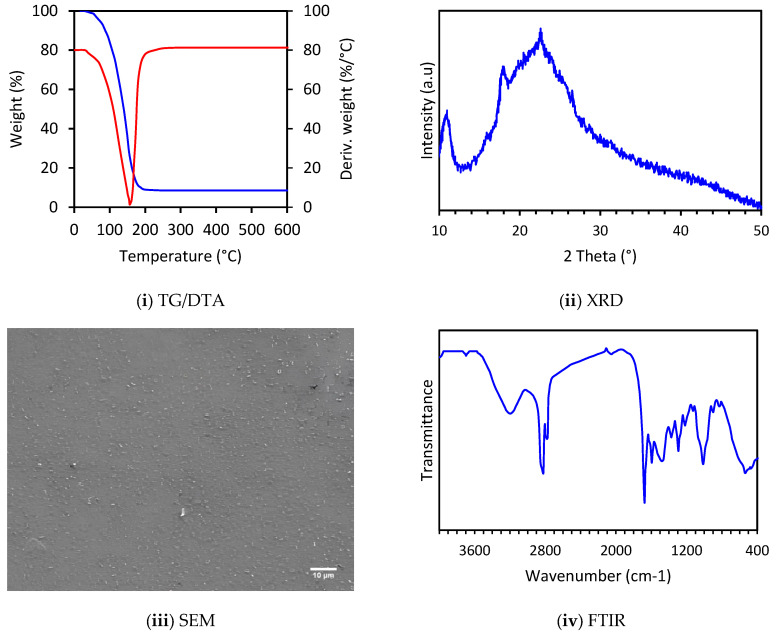
Thermal and structural analyses of the CB3C-5 biofilm (TGA-DTA: thermogravimetric analysis and differential thermal analysis; XRD: X-ray diffraction; SEM: scanning electron microscopy; FTIR: Fourier transform infrared spectroscopy).

All thermal and structural analyses are displayed in Figure 4. TGA-DTA thermal analyses were used to study the thermal stability of the CB3C-5 biofilm. A slow mass loss was observed between 100 °C and 200 °C, probably due to the evaporation of moisture and organic compounds present in the biofilm [32]. X-ray diffraction (XRD) analysis showed an amorphous structure with characteristic weakly crystallized peaks at 11.1° and 18.2°, corresponding to the hydrated and anhydrous crystallization of chitosan, respectively, and a peak at 23.5° corresponding to the trispiral structure of collagen [33,34,35]. Scanning electron microscopy (SEM) analysis revealed a rough and regular surface of the biofilm, with dense grains and concavities. In addition, the presence of flocculation and aggregation in the biofilm fluids was observed, suggesting that some reaction products exist in the biofilm [36]. Fourier transform infrared spectroscopy (FTIR) results confirm the presence of chitosan, collagen, and Calothrixin A in the biofilm structure. Specific bands were observed for each component, confirming the SEM and XRD results: at 3450 cm^−1^ for chitosan, at 1650 cm^−1^ and 1550 cm^−1^ for collagen amides, and at 2850 cm^−1^ and 1700–1650 cm^−1^ for functional groups of Calothrixin A [37,38,39].

### 2.4. CB3C-5 Biofilm Insecticidal Activity

**Figure 5:**
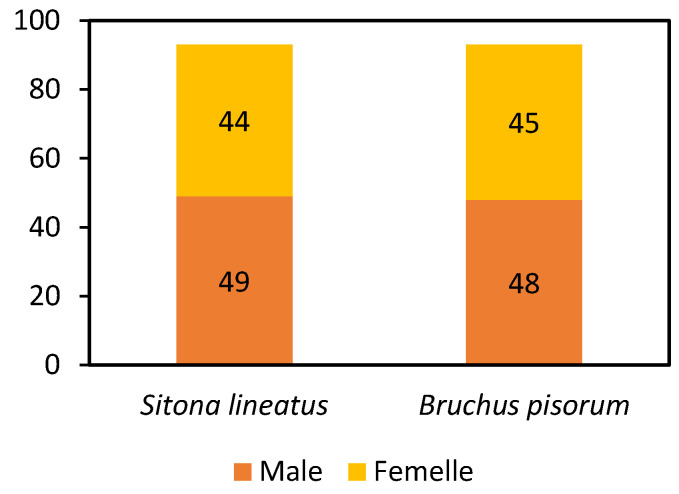
Graphical representation of mortality data from insecticide activity according to contingency table data.

The analysis of data relating to the insecticidal activity of the CB3C-5 biofilm was conducted following a rigorous methodological approach, supported by the $\chi^{2}$ test of independence, performed from the contingency table (Table 7). The two insect species, *Sitona lineatus* and *Bruchus pisorum*, were chosen for this study on insecticidal activity. The information collected, in the form of mortalities, was organized in the form of a cross-tabulation (Figure 5), which distinguishes individuals according to their sex, with the columns “Male” and “Female” representing, respectively, the males and females of the two species.

Two hypotheses were formulated to test the independence of the two categorical variables, namely the insect species and the sex. The null hypothesis (H_0_) states that the pea insect species and the sex are independent, while the alternative hypothesis (H_1_) postulates that there is a link between the two variables. Table 8 presents all the parameters calculated during the statistical analyses, including various coefficients such as Pearson’s Phi, the contingency coefficient, Cramer’s V, and other statistical indicators.

Following the values presented, the results of the tests and coefficients indicate a very weak association between the studied variables. All the coefficients are close to 0, which confirms that the variables are independent. This is also proven by the high *p*-value (1.0), which suggests the absence of a statistically significant link between these two variables.

### 2.5. Computational Analysis and Mechanism

In this study, a detailed protocol was proposed for the molecular docking of Calothrixin A with two specific proteins: the macromolecule OR5-Orco (PDB ID: 8Z9Z) and the macromolecule OBP (PDB ID: 4F7F).

The OR5-Orco complex consists of two subunits: OR5, a receptor protein that specifically binds to odorant molecules, and Orco, a co-receptor essential for stabilizing the receptor and activating the neuronal response when an odorant molecule binds to OR5. This complex allows the insect to detect specific molecules, used for orientation towards hosts or food sources [40,41]. In parallel, the Olfactory Binding Protein (OBP) also plays a key role in this system, by facilitating the transport of odorant molecules to olfactory receptors located on sensory neurons. This interaction is essential for the detection of pheromones and other chemical signals. OBPs may therefore be potential targets for insect repellents or control strategies [42,43].

**Figure 6:**
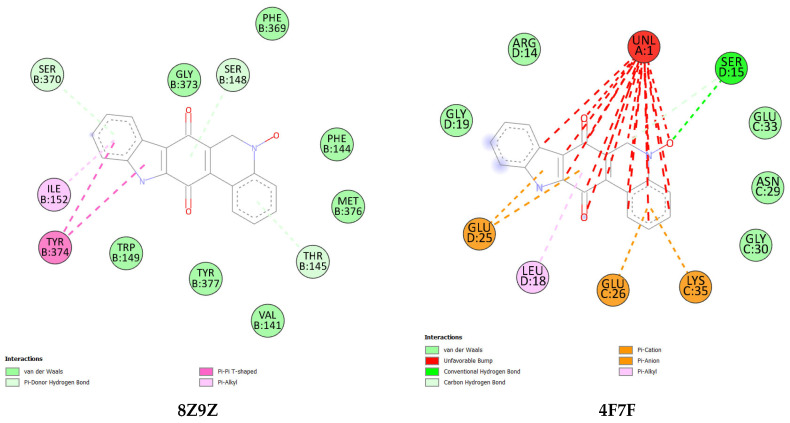
Molecular docking: interactions between the ligand (Calothrixin A) and the proteins of insecticidal activity.

The molecular docking of Calothrixin A with both proteins revealed varied interactions, as shown in Figure 6 and the thermodynamic parameters displayed in Table 9. The docking of Calothrixin A with 8Z9Z showed several types of binding interactions: three π-donor hydrogen bonds, two π − π T-shaped, one pi-alkyl, and seven van der Waals bonds. In contrast, docking with 4F7F yielded 13 unfavorable interactions, 2 π-anion, π pi-cation, 1 conventional hydrogen bond, 1 carbon conventional bond, 1 pi-alkyl, and 5 van der Waals.

The results show that the 8Z9Z complex exhibits better binding affinity, higher ligand efficiency, and a higher pKi value (7.55 vs. 5.79 for 4F7F). This suggests that 8Z9Z is a more promising ligand in terms of affinity and efficacy for the target studied.

Concerning the mechanism of action, the OR5-Orco complex plays a fundamental role in the detection of odorous molecules, allowing the insect to orient itself towards its hosts or food sources. The 8Z9Z ligand, with its stronger affinity for OR5-Orco, could disrupt this olfactory detection, by interfering with neuronal activation [44]. These results open the way to new strategies for the control of insect pests by targeting this olfactory complex, thus reducing their ability to locate food and hosts, and offering a means of ecological pest control.

## 3. Discussion

This study developed and refined an insecticidal biofilm based on marine products, such as Calothrixin A, collagen, and chitosan, for the protection of pea seeds against the pests *Sitona lineatus* and *Bruchus pisorum*. The results confirm that Calothrixin A plays a key role in the insecticidal efficacy of the biofilm. A high concentration of collagen and chitosan reduced this activity, probably by limiting the diffusion of the active ingredient. Thus, the controlled release of Calothrixin A proved to be decisive in optimizing the insecticidal effect. The analysis of the mathematical models showed that the studied factors and their interactions had no significant impact on the pLD50 values for *Sitona lineatus* and *Bruchus pisorum*, although the predictive model for *Sitona lineatus* demonstrated better accuracy. The study of Calothrixin A desorption highlighted that diffusion parameters directly influence the insecticidal efficacy of the biofilm. The CB3C-5 biofilm was found to be optimal, with high diffusion, retention coefficient, and desorption flux values, reflecting a rapid and efficient release of the active ingredient. Strong correlations were observed between these parameters and the insecticidal activity, highlighting the importance of precise diffusion control to maximize the efficacy of the biofilm. Furthermore, the physicochemical characterization of the CB3C-5 biofilm revealed a thin and homogeneous structure, partial solubility, efficient moisture management, and sufficient mechanical robustness. These properties are essential to ensure practical application in agricultural environments. FTIR analysis confirmed the homogeneous integration of Calothrixin A into the biofilm matrix, thus validating the chemical stability of the active compound. The computational approach revealed that Calothrixin A interacts more efficiently with the OR5-Orco complex, which is involved in insect olfactory detection. This mode of action could disrupt their orientation toward food and host sources, providing an alternative pest control strategy. These results reinforce the idea that the developed biofilm represents a viable and effective solution for the protection of pea seeds against insect pests.

The results of this study are distinct from those of previous studies that have explored similar strategies. Anh and Dumri (2017) [45] developed a hybrid film that absorbs organophosphate insecticides, but their approach relied on adsorption rather than active insecticidal action. Similarly, Bejan et al. (2018) [46] designed chitosan-based luminescent hydrogels, but their application was more oriented toward optical devices than pest control. Rapichai et al. (2021) [47] developed a device for the detection of OP insecticides, based on a chitosan film immobilizing a specific enzyme, illustrating a complementary analytical approach to the present study. Missaoui et al. (2023) [48] developed insecticide films based on clay nanocomposites, demonstrating efficacy against Tribolium castaneum, a species different from those targeted here. Finally, Kandeh et al. (2023) [49] fabricated an adsorbent nanocomposite for the microextraction of pesticides, illustrating another perspective on the removal of chemical contaminants.

The demonstrated efficacy of the CB3C-5 biofilm in this study is based on the optimization of the diffusion of Calothrixin A, which was not specifically explored in the mentioned works. The computational approach also identified an innovative mode of action through interaction with the OR5-Orco complex, which constitutes a notable advancement compared to traditional insecticide methods.

The results obtained have important implications on theoretical and practical levels. From a theoretical point of view, this study has allowed us to better understand the influence of diffusion parameters on the insecticidal efficacy of a biofilm, as well as the molecular interactions of Calothrixin A with the olfactory receptors of insect pests. This knowledge could be used to design new targeted biological control strategies. On a practical level, the CB3C-5 biofilm represents a promising alternative to chemical insecticides, thus reducing the environmental impact of conventional treatments. Its efficacy against *Sitona lineatus* and *Bruchus pisorum* could promote its adoption in agricultural protection. In addition, its mechanical robustness and chemical stability guarantee prolonged application in real conditions.

Future studies could focus on evaluating the biofilm under real conditions to test its durability and effectiveness in the field. An analysis of its ecological impact, particularly on the biodiversity of environmental components, would also be necessary. Furthermore, the optimization of the biofilm formulation to broaden its spectrum of action to other harmful insects could be considered.

## 4. Material and Methods

### 4.1. Reagents and Marine Molecules

The chemical reagents used in this study were purchased from high-purity suppliers, ensuring their quality and suitability for the experimental objectives of this research.

Calothrixin A was isolated from Calothrix extracts following the procedure described by Rickards et al. (1999) [50]. Cells of the bioactive Calothrix strains were freeze-dried to obtain dry biomass and then extracted using dimethyl sulfoxide (DMSO) and ethyl acetate (AcOEt) under Soxhlet conditions, resulting in extracts rich in bioactive compounds. These extracts were then subjected to column chromatography using solvents of increasing polarity, thus separating the different constituents present to isolate pure Calothrixin A. (Spectral information: 1H NMR (600 MHz, DMSO-d6): δ 9.68 (bd, 1H, CH-I), 7.98 (m, 1H, CH-2), 7.96 (m, 1H, CH-3), 8.60 (bd, 1H, CH-4), 8.88 (s, 1H, CH-6), 8.11 (bd, 1H, CH-8), 7.37 (bt, 1H, CH-9), 7.44 (dt, 1H, CH-10), 7.60 (bd, 1H, CH-I1), 13.2 (b, 1H, NH); 13C NMR (151 MHz, DMSO-d6): δ 128.2 (CH-I), 132.0 (CH-2), 132.1 (CH-3), 119.2 (CH-4), 143.1 (C-4a), 131.9 (CH-6), 130.0 (C-6a), 178.4 (C-7), 115.2 (C-7a), 123.6 (C-7b), 122.1 (CH-8), 124.6 (CH-9), 127.1 (CH-10), 114.2 (CH-I1), 138.4 (C-I1a), 139.0 (C-12a), 177.9 (C-13), 122.1 (C-13a), 126.9 (C-13b); MS (EI): *m*/*z* = 315.07 [M + H]^+^.)

The preparation of chitosan was carried out according to the method described by El Knidri et al. (2018) [51]. The exoskeletons of *Penaeus semisulcatus* (green tiger shrimp) from the Gulf of Tadjoura, Djibouti (11°45′40.590″ N, 42°53′2.472″ E), were cleaned, dried, and ground into a fine powder. This powder was treated with a dilute solution of 2 M hydrochloric acid (HCl) in a solid/solvent ratio of 1:15. The mixture was stirred at room temperature for 3 h to remove mineral salts such as calcium carbonate and calcium chloride. After this period, the residue was filtered, washed with demineralized water until neutral, and dried at 50 °C for 3 days. The demineralized residue was then treated with a solution of 2.56 M sodium hydroxide (NaOH) in a solid/solvent ratio of 1:10. The mixture was stirred at 80 °C for 45 min to remove proteins. After this step, the residue was filtered, washed several times with deionized water to remove excess NaOH, and dried at 50 °C for 3 days. The purified chitin was then treated with 50% sodium hydroxide (NaOH) solution in a solid/solvent ratio of 1:10. The mixture was heated at 100 °C for 2 h to remove acetyl groups, thereby transforming the chitin into chitosan. After the reaction, the material was washed several times with deionized water until neutral and dried at 50 °C for 3 days. (Spectral information: ^1^H NMR (500 MHz, DMSO-d6): δ 4.8 (H1), 3.1 (H2), 3.6–3.8 (H3, H4, H5, H6), 2.0 (N-acétyl); ^13^C NMR (125 MHz, DMSO-d6): δ 100 (C1), 60 (C2), 73 (C3), 80 (C4), 78 (C5), 63 (C6); MS (EI): *m*/*z* = 40–45, 59, 67, 80, 94 (peaks most intensive).

Collagen preparation was performed according to the method described by Mao et al. (2024) [52]. The skin of *Priacanthus tayenus*, from the Gulf of Eden in Djibouti ((11°49′9.498″ N, 43°31′23.696″ E), was cut into 1 cm^3^ squares, after removing the flesh and scales. The samples were immersed in a 0.1 M NaOH solution, with a liquid/solid ratio of 1:25, and left at 5 °C for 24 h to defat. After neutralization with deionized water, the pieces were treated with a 5% Na_2_CO_3_ solution (liquid/solid ratio of 1:25) at 4 °C for 24 h to purify. Collagen extraction was performed by mixing the skin with 1M acetic acid solution (liquid/solid ratio of 1:25), followed by grinding for 30 s, homogenization at 10,000 rpm for 2 min, ultrasonication at 0.5 kW for 10 min, and then static extraction at 5 °C for 48 h. After centrifugation at 10,000× *g* for 15 min at 5 °C, the supernatant was salted with NaCl to a concentration of 2M and left at 5 °C overnight to allow the aggregation of flocculent precipitates. After a second centrifugation, the obtained white precipitates were dissolved in 0.5 M acetic acid solution and dialyzed against deionized water for 3 days, with two solution changes every 12 h. Finally, the dialyzed solutions were frozen and lyophilized to obtain collagen powder.

### 4.2. Preparation of Composite Biofilms

The preparation method of the composite biofilms (CB3C) was carried out in several steps. First, 20 g of freeze-dried collagen powder was dissolved in water and stirred at 50 °C for 30 min, thereby forming a 1–2% (*w*/*v*) collagen solution. At the same time, 20 g of chitosan was dissolved in 0.5 mol/L acetic acid solution and then stirred at 50 °C for 30 min to obtain a 1–2% (*w*/*v*) chitosan solution. The two solutions were then mixed at a volumetric ratio of 1:1, and after stirring, the solution was heated in a water bath at 55 °C for 30 min to induce crosslinking between collagen and chitosan. Calothrixin A, at a concentration of 0.1% to 0.5% (*w*/*v*), was added to the solution, along with 0.25% (*w*/*v*) glycerin, to improve film plasticity. Homogenization was performed using a high-speed dispersant (Silverson, England) at 10,000 rpm for 2 min, and then bubbles were removed. The film-forming solutions were then cast onto 20 cm diameter polyethylene plates and dried for 24 h in an electrothermal incubator at 25 °C. After drying, the films were placed in a desiccator at 70% relative humidity for 24 h to equilibrate the humidity. Finally, the films were carefully removed from the plates once humidity equilibrium was reached [53,54,55].

### 4.3. Insecticide Test

Adults of *Sitona lineatus* and *Bruchus pisorum* were obtained from the breeding stock maintained at the laboratory of the Institut de Recherche Médicinale de Djibouti (IRM, CERD Djibouti), at a constant temperature of 25 ± 2 °C. These insects were placed in glass containers containing dried peas, with the containers closed with a non-woven fabric to maintain an environment conducive to their reproduction. For each bioassay, 2 cm × 2 cm plates of composite biofilm were placed in a Petri dish (Φ=90 mm×h=15 mm), which was then incubated at 25 °C for 2 h. After this period, 10 insects of each species were transferred to the box containing the biofilms, and the test was maintained in the dark at 28 ± 2 °C [56].

Mortality rates (M%) were recorded after a 7-day incubation period. The corrected mortality in treated insects is expressed according to the following formula [56,57]:

$$\mathrm{CM}\%=\left(\frac{MOI-MOC}{100-MOC}\right)\times 100$$

- CM% = corrected mortality.
- *MOI* = mortality observed in insects (%).
- *MOC* = mortality observed in controls (%).

The determination of the median lethal dose (LD50) was performed through linear interpolation from the curves obtained, correlating the percentage of corrected mortality to the logarithm of the concentrations of the biofilms tested. For technical reasons, the natural logarithm of this function, denoted as pLD50=Ln(LD50), was adopted to evaluate the insecticidal efficacy. Thus, the higher the value of pLD50, the more significant the consideration of insecticidal activity [57].

In addition, a complementary study was conducted to explore the impact of sex and insect species on the effects of the biofilm insecticide (CB3C-5) after optimization. For this test, 100 insects of each species (50 males and 50 females) were placed in a 20 mL jar containing 5 composite biofilm plates (2 cm × 2 cm), also prepared according to the method described above. This second section aims to provide additional information on the mechanisms of action of insecticidal biofilms, particularly concerning the regeneration of insects after exposure [58].

Each experiment was performed in triplicate to ensure the reliability of the results.

### 4.4. Analytical Characterization of Biofilm

The composite biofilm (CB3C-5) was characterized physicochemically using various analytical techniques. Film thickness was measured using a MITUTOYO QuantuMike micrometer (Mitutoyo Corporation, Kawasaki, Japan), with a measuring range of 1 to 2 inches, Digimatic model, IP65. This method allowed thickness to be assessed by pinching the material between two flat disks, after calibrating the instrument to zero. The moisture absorption (MA) of the biofilms was determined by drying 2cm×2cm samples in an oven at 80 °C and 70% relative humidity and then measuring the mass change. Water solubility (WS) was assessed by immersing biofilm strips 2cm×2cm in 50 mL of distilled water at 30 °C for 24 h. After drying at 105 °C to constant weight, solubility was calculated based on mass loss. The opacity (OP) of the biofilms was measured spectrophotometrically, assessing absorbance at a specific wavelength. Water vapor permeability (WVP) was determined by measuring the vapor transmission rate through the material, using a Systech Lyssy L80-6000 Water Vapor Permeation Analyzer (Saicheng, Shandong, China).

The mechanical properties of the biofilms were assessed by measuring tensile strength (TS) and elongation at break (EAB) using an ASTM GT-UA03 single-column tensile testing machine (Barrus, Oxfordshire, UK).

Thermal analyses were performed by thermogravimetric analysis and differential thermal analysis (TGA-DTA: DTG-60H system, Shimadzu, Kyoto, Japan). TGA was used to measure the mass changes in the biofilms as a function of temperature to study their thermal stability and decomposition. DTA measured the temperature difference between the films and a reference, subjected to the same thermal program, to identify thermal transitions.

Structural analyses were performed by X-ray diffraction (XRD: D8 Advance diffractometer with Cu Kα radiation at 1.54 Å, Bruker Corporation, Billerica, MA, USA), scanning electron microscopy (SEM: JSM-6700F, JEOL Ltd., Tokyo, Japan), and Fourier transform infrared spectroscopy (FTIR: Vertex 70, Bruker Corporation, Billerica, MA, USA). XRD identified the crystalline structures of the films by measuring the angles and intensities of the diffracted X-rays. SEM provided detailed images of the surface of the films, revealing their morphology and topography. FTIR analyzed the molecular vibrations of the films by measuring the absorption of infrared light, providing information on the chemical bonds present.

### 4.5. Calothrixin A Desorption Modeling

**Figure 7:**
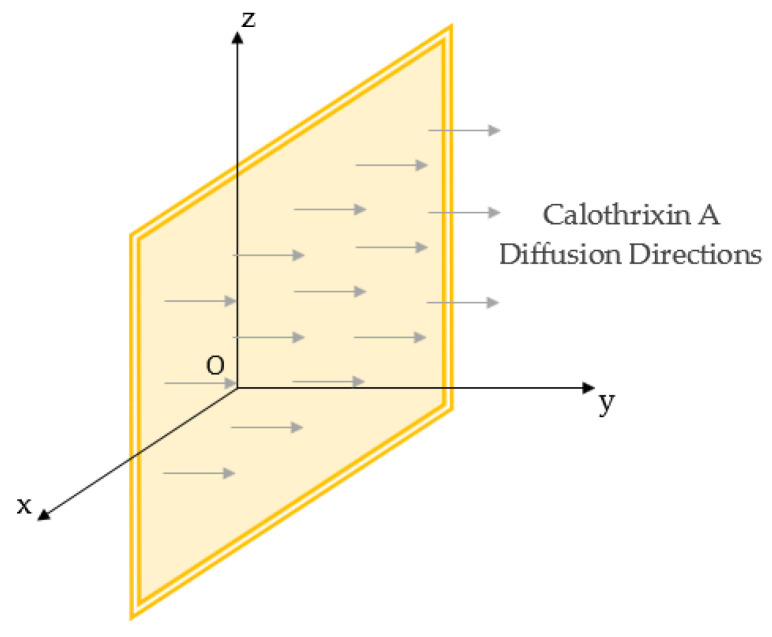
Diffusion directions following (Oy) in a square sheet according to Fick’s second law.

In the study of the desorption of Calothrixin A through biofilms modeled as square films of dimensions 2cm×2cm, a mathematical approach was implemented to model this process and to determine the diffusion-related parameters. This method is based on the following fundamental assumptions:

- The biofilm is modeled as a thin, flat sheet of square shape, with one-dimensional diffusion occurring along the (Oy) axis (Figure 7).
- Diffusion occurs under transient conditions, meaning that the concentration of Calothrixin A decreases over time.
- The diffusivity of Calothrixin A is assumed to be constant throughout the diffusion process.
- At the beginning of desorption, the concentration of Calothrixin A throughout the biofilm is uniform and constant.

The desorption process is modeled by the Fick diffusion equation [59], which describes the variation in the concentration C(y,t) of the liquid as a function of time t and position y in the biofilm sheet. The diffusion equation used is

(1) $$\frac{\partial C}{\partial t}=D\frac{\partial^{2}C}{\partial y^{2}}$$

where

- C(y,t) is the concentration of Calothrixin A at a position y  and at a time t;
- D is the diffusivity of the liquid in the film;
- y is the position in the film;
- t  is the time.

### 4.6. Initial and Boundary Conditions:

- Initial condition: At t=0, the concentration C(y,0) is uniform across the entire e thickness of the film:

(2) $$C(y,0)=C_{0}\mathrm{for}\;0\leq y\leq e$$

where $C_{0}$ is the initial concentration, and L is the thickness of the film.

- Boundary condition: At the edge of the film sheet (one side only), the concentration of the liquid becomes zero during desorption:

(3) C(L,t)=0for t>0

which implies that all of the liquid is desorbed at the surface of the film.

***Diffusion solution:***

The solution to this diffusion equation can be expressed as an infinite series, giving the concentration C(y,t) at each position y and each time t in the biofilm. This solution is formulated as follows:

(4) $$C\left(y,t\right)=C_{0}-C^{*}+\sum_{m=1}^{\infty }{\left(-1\right)}^{m}.sin\left((2m+1)\pi y\right).exp\left(-D.\frac{{\left(2m+1\right)}^{2}}{e^{2}}.\pi^{2}.t\right)$$

where

- $C_{0}$ is the initial concentration of Calothrixin A.
- $C^{*}$ is the saturation concentration of the liquid in the biofilm.
- *D* is the diffusion coefficient of the liquid.
- *y* is the position along the axis (*Oy*).

The total mass desorbed at a time t can be obtained by integrating the concentration C(y,t) over the entire thickness of the biofilm. This integration is carried out over the interval 0≤y≤L:

(5) $$M_{t}=\int_{0}^{e}S.C\left(y,t\right)dy\;$$

where S represents the surface area of the biofilm. In taking only the first term of the infinite series for the approximation, this expression can be simplified as follows:

(6) $$\frac{M_{t}}{M_{0}}=exp\left(\frac{{-D.\pi }^{2}.t}{e^{2}}\right)$$

where $M_{0}$ is the initial mass of Calothrixin A in the biofilm.

After numerical modeling using the MATLAB computer program (R2019a, 2019), the main parameters were predicted:

- The diffusivity D can be determined from the equation of the total desorbed mass by plotting the curve $Ln(M_{t}/M_{0})$ as a function of time t. The slope of this curve allows the diffusivity D to be determined.
- The evaporation constant K is related to the diffusivity D by the following relation:

(7) $$K=\frac{\pi^{2}.D}{e^{2}}$$

- The evaporation rate F is the initial desorption rate and can be calculated from the derivative of the desorbed mass with respect to time ttt at time t=0. This derivative can be approximated from the expression of $M_{t}$:

(8) $$F=F_{0}=\frac{1}{S}.{lim}_{t\to 0}\frac{dM}{dt}$$

### 4.7. Computational Approach

Molecular docking has been used as an essential method to predict the interaction between a target molecule and a receptor protein, thus facilitating the understanding of insecticidal mechanisms. In this study, a detailed protocol was proposed for the docking of Calothrixin A with two specific proteins: the OR5-Orco complex (PDB ID: 8Z9Z), an olfactory receptor in insects, and a small protein involved in the olfactory system of insects (PDB ID: 4F7F). Calothrixin A was downloaded from the PubChem database, prepared by optimizing its geometry and adding the necessary hydrogen atoms. The structures of the proteins 8Z9Z and 4F7F were extracted from the Protein Data Bank (PDB), and then prepared by optimizing their geometry, adding hydrogen atoms, and assigning partial charges. The active sites of the proteins were identified by examining their three-dimensional structure.

Docking grids were defined around the active sites using AutoDockTools (V1.5.6, 2015), with dimensions and resolution adapted to adequately cover the target sites. Docking simulations were performed using AutoDock Vina, with optimized parameters to generate a sufficient number of poses and ensure the convergence of results. The results were analyzed with Discovery Studio, allowing us to visualize the interactions between Calothrixin A and the residues of the active sites of the proteins. Hydrogen bonds, hydrophobic interactions, and electrostatic contacts were highlighted. The obtained binding scores were evaluated to identify the most favorable configurations, and the results were compared with available experimental data to validate the predictions. Additional simulations were considered to confirm the predicted interactions, and the results were interpreted in terms of potential mechanisms of action of Calothrixin A in the insect olfactory system [60,61,62].

### 4.8. Data Statistics

#### 4.8.1. Experimental Design

To optimize biofilm mortality, a full factorial experimental design approach was adopted. Three main factors were selected:

- Collagen concentration: 1% (−1) and 2% (+1) (*w*/*v*);
- Chitosan concentration: 1% (−1) and % (+1) (*w*/*v*);
- Calothrixin A concentration: 0.1% (−1) and 0.5% (+1) (*w*/*v*).

Each factor was studied at two levels, coded, respectively, by +1 for high level and −1 for low level. The total number of tests performed was determined based on the number of factors, according to the formula $2^{k}$, where k represents the number of factors studied. Thus, for three factors, a total of eight tests were performed.

The analysis of the results was performed using a linear model including main effects and interactions up to the third order [63], according to the following equation:

(9) $$M(\%)={µ}_{0}+\sum_{i=1}^{n}{µ}_{i}X_{i}+\sum_{i=1}^{n}\sum_{j=1}^{n-1}{µ}_{ij}X_{i}X_{j}+\sum_{i=1}^{n}\sum_{j=1}^{n-1}\sum_{k=1}^{n-2}{µ}_{ijk}X_{i}X_{j}X_{k}$$

- $\mu_{0}$: mean of mortality;
- $\mu_{i}$: main effects for each factor;
- $\mu_{ij}$: effects of second-order interactions;
- $\mu_{ijk}$: effect of third-order interactions.

This methodology allowed us to evaluate not only the individual effect of each factor on insect mortality, but also the potential interactions between these factors [64]. The use of a full factorial design ensured a robust optimization of the biofilm preparation process, providing a comprehensive view of the possible combinations of the factors studied.

#### 4.8.2. Correlations

To analyze the relationships between the mortality of the two species according to sex, the Pearson $\chi^{2}$ test was used [65]. This statistical test evaluates the independence between two categorical variables by comparing the observed frequencies with the expected frequencies in a contingency table. The statistical decision was based on the comparison of the calculated $\chi^{2}$ value with the critical value from the $\chi^{2}$ distribution table, according to the degrees of freedom and the chosen significance threshold (generally α=0.05). If the calculated value exceeded the critical value, the null hypothesis of independence was rejected, suggesting a significant association between the studied variables. Also, other association parameters were calculated.

In addition, a principal component analysis (PCA) was performed to explore the relationships between the adsorption parameters and the calculated descriptors [66]. PCA is a dimensionality reduction technique that transforms correlated variables into a set of uncorrelated variables, called principal components. This approach facilitated the interpretation of the data by identifying the major axes of variation and providing a graphical representation of the correlations.

#### 4.8.3. Uncertainties

The statistical analysis of the results was performed using the XLSTAT tool (version 2016), integrated into Microsoft Excel 13. The data were presented as means accompanied by their associated uncertainties, with a significance threshold set at 5% for each test [67].

## 5. Conclusions

This study investigated the development and optimization of an insecticidal biofilm based on Calothrixin A, collagen, and chitosan for pea seed protection. The objective was to maximize the insecticidal activity and desorption of Calothrixin A by analyzing the influence of diffusion factors on biofilm efficacy. Optimization revealed that Calothrixin A had a significant impact on insecticidal activity, while high concentrations of collagen and chitosan reduced this activity. The results also showed that the CB3C-5 biofilm exhibited rapid diffusion, high retention, and efficient release of the substance, optimizing its efficacy against Sitona lineatus and Bruchus pisorum. Statistical analysis confirmed that insect species and sex variables were independent, which did not affect treatment efficacy. The CB3C-5 biofilm was characterized by physical, mechanical, and thermal properties optimized for agricultural application. Significant results were obtained, including a tensile strength of 17.4 MPa and efficient water vapor permeability. In the computational approach, docking revealed that Calothrixin A interacts more effectively with the OR5-Orco complex, disrupting insect olfactory detection and reducing their ability to locate their hosts. This paves the way for new strategies to control insect pests by targeting this olfactory complex. Future perspectives of this research include further exploring the impact of environmental factors on biofilm effectiveness and improving prediction models to optimize insecticidal action. These results pave the way for innovative pest control strategies by targeting olfactory mechanisms and they provide a solid foundation for ecological and sustainable agricultural applications.

## Figures and Tables

**Table 1:** Insecticidal activity of optimized biofilm preparations.

	Factor 1	Factor 2	Factor 3	*Sitona* *lineatus*	*Bruchus* *pisorum*
Biofilm	A: Collagen (%)	B: Chitosan (%)	C: Calothrixin A (%)	pLD50	pLD50
CB3C−1	−1	−1	−1	4.17	4.26
CB3C−2	1	−1	−1	4.12	4.32
CB3C−3	−1	1	−1	4.12	4.28
CB3C−4	1	1	−1	4.08	4.22
CB3C−5	−1	−1	1	4.56	4.59
CB3C−6	1	−1	1	4.44	4.52
CB3C−7	−1	1	1	4.41	4.5
CB3C−8	1	1	1	4.36	4.51

**Table 2:** Mathematical models used to predict pLD50.

Model	F Value	*p*-Value	Significance
pLD50 (SL) = 4.28 − (0.03 × A) − (0.04 × B) + (0.16 × C) + (0.01 × AB) − (0.01 × AC) − (0.02 × BC) + (0.01 × ABC)	85.22	0.08	Not significant
pLD50(BP) = 4.40 − (0.01 × A) − (0.02 × B) + (0.13 × C) − (0.01 × AB) − (0.01 × AC) − (0.01 × BC) + (0.03 × ABC)	4.68	0.34	Not significant

**Table 3:** Statistical parameters of pLD50.

Parameter	*Sitona lineatus (*SL*)*	*Bruchus pisorum (*BP*)*
Mean of pLD50	4.28	4.40
Standard deviation	0.02	0.07
Coefficient of variation (%)	0.49	1.61
R^2^	0.998	0.966
Adjusted R^2^	0.986	0.760
Predicted R^2^	0.875	−1.201
A_deq_ Precision	23.43	4.84

**Table 4:** Desorption parameters of Calothrixin A following diffusion.

Biofilm	D (×10^6^) (mm^2^ day^−1^)	K (×10^3^)	F (×10^5^) (g day^−1^ cm^−2^)
CB3C-1	1.15	0.79	0.50
CB3C-2	0.76	0.52	2.00
CB3C-3	1.53	1.05	1.73
CB3C-4	0.15	0.10	0.13
CB3C-5	3.82	2.62	3.00
CB3C-6	2.67	1.83	2.00
CB3C-7	1.91	1.31	2.38
CB3C-8	0.76	0.52	2.75

**Table 5:** Correlation matrix of desorption with insecticidal activity.

Variables	D	K	F	pLD50 (SL)	pLD50 (BP)
D	1				
K	1.000	1			
F	0.603	0.603	1		
pLD50 (SL)	0.829	0.828	0.755	1	
pLD50 (BP)	0.741	0.740	0.867	0.970	1

**Table 6:** Physical and mechanical properties of CB3C-5 biofilm.

**Biofilm thickness**	e = 0.12 ± 0.02 mm
Moisture absorption	MA = 3.9 ± 0.2 %
Water solubility	WS = 26.5 ± 2.4 %
Opacity	OP = 1.1 ± 0.1 A mm^−1^
Water vapor permeability	WVP = 1.8 ± 0.3 g m^−1^ Pa^−1^ s^−1^
Tensile strength	TS = 17.4 ± 1.5 MPa
Elongation at break	EB = 122.7 ± 5.1 %

**Table 7:** Contingency table.

Insects	Male	Female
*Sitona lineatus*	49	44
*Bruchus pisorum*	48	45

**Table 8:** Chi-square test parameters between insect species and sex.

Coefficient	Value
Pearson’s Phi	0.011
Contingency Coefficient	0.011
Cramér’s V	0.011
Tschuprow’s T	0.011
Goodman and Kruskal’s rate	0.000
Cohen’s Kappa	0.011
Yule’s Q	0.022
Yule’s Y	0.011

**Table 9:** Thermodynamic parameters of molecular docking.

Parameter	8Z9Z	4F7F
pKi	7.55	5.79
Binding affinity (kcal mol^−1^)	−10.3	−7.9
Ligand efficiency (kcal mol^−1^)	0.429	0.329

## Data Availability

Data are contained within the article.
